# FASt single‐breathhold 2D multislice myocardial T_1_ mapping (FAST1) at 1.5T for full left ventricular coverage in three breathholds

**DOI:** 10.1002/jmri.26869

**Published:** 2019-07-24

**Authors:** Li Huang, Radhouene Neji, Muhummad Sohaib Nazir, John Whitaker, Phuoc Duong, Fiona Reid, Filippo Bosio, Amedeo Chiribiri, Reza Razavi, Sébastien Roujol

**Affiliations:** ^1^ The School of Biomedical Engineering and Imaging Sciences, Faculty of Life Sciences and Medicine King's College London London UK; ^2^ MR Research Collaborations Siemens Healthcare Limited Frimley UK; ^3^ School of Population Health and Environmental Sciences, Faculty of Life Sciences and Medicine King's College London London UK

**Keywords:** myocardial tissue characterization, T1 mapping, multislice, slice‐selective, MOLLI, inversion recovery

## Abstract

**Background:**

Conventional myocardial T_1_ mapping techniques such as modified Look–Locker inversion recovery (MOLLI) generate one T_1_ map per breathhold. T_1_ mapping with full left ventricular coverage may be desirable when spatial T_1_ variations are expected. This would require multiple breathholds, increasing patient discomfort and prolonging scan time.

**Purpose:**

To develop and characterize a novel FASt single‐breathhold 2D multislice myocardial T_1_ mapping (FAST1) technique for full left ventricular coverage.

**Study Type:**

Prospective.

**Population/Phantom:**

Numerical simulation, agarose/NiCl_2_ phantom, 9 healthy volunteers, and 17 patients.

**Field Strength/Sequence:**

1.5T/FAST1.

**Assessment:**

Two FAST1 approaches, FAST1‐BS and FAST1‐IR, were characterized and compared with standard 5‐(3)‐3 MOLLI in terms of accuracy, precision/spatial variability, and repeatability.

**Statistical Tests:**

Kruskal‐Wallis, Wilcoxon signed rank tests, intraclass correlation coefficient analysis, analysis of variance, Student's *t*‐tests, Pearson correlation analysis, and Bland–Altman analysis.

**Results:**

In simulation/phantom, FAST1‐BS, FAST1‐IR, and MOLLI had an accuracy (expressed as T_1_ error) of 0.2%/4%, 6%/9%, and 4%/7%, respectively, while FAST1‐BS and FAST1‐IR had a precision penalty of 1.7/1.5 and 1.5/1.4 in comparison with MOLLI, respectively. In healthy volunteers, FAST1‐BS/FAST1‐IR/MOLLI led to different native myocardial T_1_ times (1016 ± 27 msec/952 ±22 msec/987 ± 23 msec, *P* < 0.0001) and spatial variability (66 ± 10 msec/57 ± 8 msec/46 ± 7 msec, *P* < 0.001). There were no statistically significant differences between all techniques for T_1_ repeatability (*P* = 0.18). In vivo native and postcontrast myocardial T_1_ times in both healthy volunteers and patients using FAST1‐BS/FAST1‐IR were highly correlated with MOLLI (Pearson correlation coefficient ≥0.93).

**Data Conclusion:**

FAST1 enables myocardial T_1_ mapping with full left ventricular coverage in three separated breathholds. In comparison with MOLLI, FAST1 yield a 5‐fold increase of spatial coverage, limited penalty of T_1_ precision/spatial variability, no significant difference of T_1_ repeatability, and highly correlated T_1_ times. FAST1‐IR provides improved T_1_ precision/spatial variability but reduced accuracy when compared with FAST1‐BS.

**Level of Evidence:** 1

**Technical Efficacy:** Stage 3

J. Magn. Reson. Imaging 2020;51:492–504.

ALTERATION OF NATIVE MYOCARDIAL T_1_ times has been observed in the presence of a variety of heart diseases such as acute and chronic myocardial infarction, myocarditis, amyloidosis, or Anderson–Fabry disease.^1^ Myocardial T_1_ mapping techniques enable pixelwise quantification of myocardial T_1_ times,^2^ which has promising value for diagnosis and prognosis.^1^

The desired spatial coverage of myocardial T_1_ mapping (single‐slice vs. multislice vs. full left ventricular [LV] coverage) may depend on the cardiac conditions, as stated by an expert consensus statement.^1^ Full LV coverage may be beneficial when spatial variations in LV wall thickness and/or fibrosis are expected, such as in the presence of hypertrophic cardiomyopathy^3^ or chronic myocardial infarction.^4^, ^5^

Several methods have been proposed for myocardial T_1_ mapping, including techniques based on inversion,^2^, ^6^ saturation,^7^ or hybrid preparation pulses.^8^ In these approaches, multiple images with different T_1_‐weightings are acquired and fit in a pixelwise manner to a physical model of the MR signal evolution.^2^ Inversion recovery (IR)‐based approaches such as modified Look–Locker inversion recovery (MOLLI)^2^ are commonly used due to their high precision, reproducibility, and map quality.^9^, ^10^, ^11^, ^12^ In these techniques, multiple (usually 7–13) 2D single‐shot electrocardiogram (ECG)‐triggered images of the same slice are acquired at different inversion times (TIs) in a single breathhold and used to generate one T_1_ map. In these conditions, myocardial T_1_ mapping with full LV coverage requires repeated breathheld acquisitions, each for one slice, thus increasing patient discomfort, prolonging scan time, and resulting in potential slice misalignment if 3D processing is necessary.

3D or advanced 2D multislice techniques can be used to achieve native myocardial T_1_ mapping with full LV coverage.^13^, ^14^, ^15^, ^16^, ^17^, ^18^, ^19^, ^20^ 3D breathheld myocardial T_1_ mapping approaches may need to compromise between spatial resolution and/or artifact level due to limited breathhold duration and limited acquisition window within the cardiac cycle.^13^, ^18^ On the other hand, 3D^14^, ^15^, ^17^, ^19^, ^20^ and 2D multislice^16^ free‐breathing myocardial T_1_ mapping require long scan times and advanced motion correction strategies, which can result in reduced map quality and increased intersegment variability compared with standard breathheld techniques such as MOLLI.^17^

In this work, we sought to develop and characterize a novel FASt single‐breathhold 2D multislice myocardial T_1_ mapping (FAST1) for myocardial T_1_ mapping with full LV coverage in three breathholds at 1.5T.

## Materials and Methods

### *Pulse Sequence*

**Figure 1:**
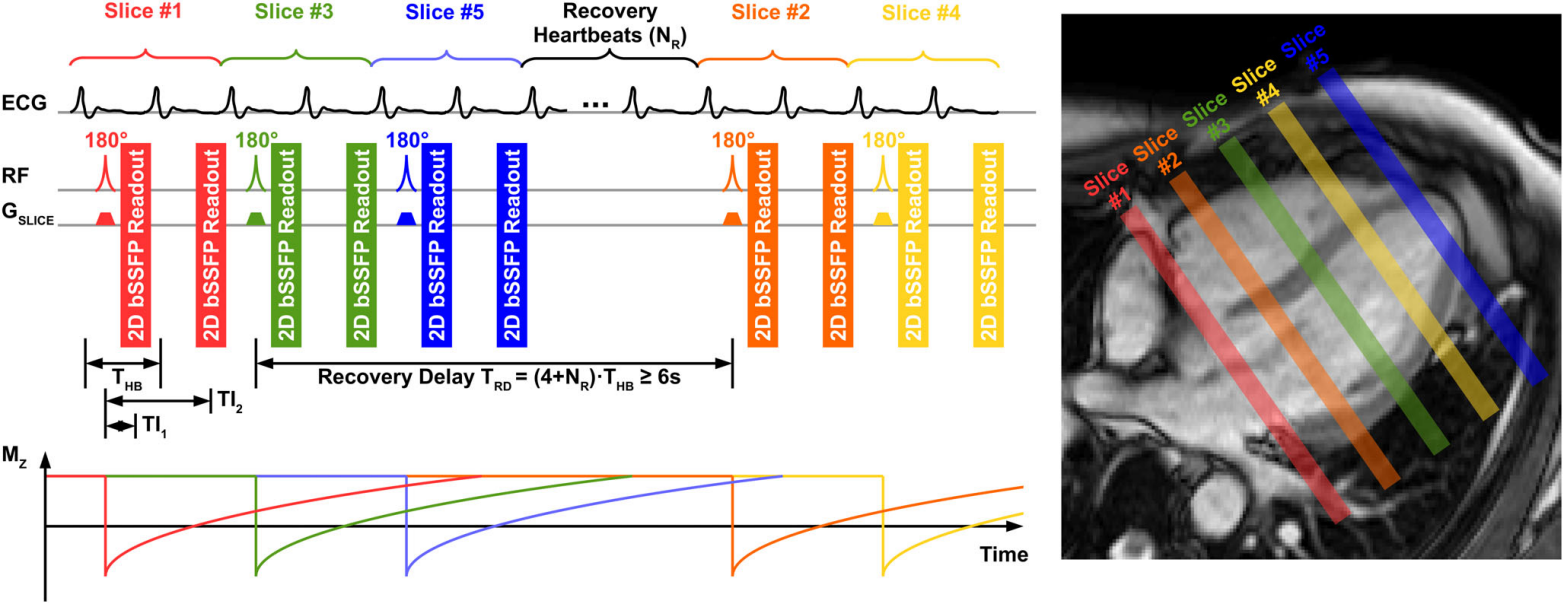
FAST1 sequence diagram and acquisition scheme. Five T_1_ maps are acquired in one breathhold, each based on a two‐heartbeat imaging block including a slice‐selective inversion pulse and the acquisition of two ECG‐triggered single‐shot images. Minimal inversion time is used for each imaging block to reduce the impact of cardiac motion between the slice‐selective inversion pulse and the first image acquisition. An inversion slice thickness larger than the imaging slice thickness is used to minimize the impact of cardiac motion. Recovery heartbeats are introduced between the third and fourth imaging blocks to minimize potential slice crosstalk between the slice‐selective inversion pulses. In the physiological HR range (50–110 bpm), the corresponding nominal breathhold duration is 9–13 sec.

The FAST1 pulse sequence diagram is illustrated in Fig. 1, where five T_1_ maps are acquired in one breathhold. A slice‐selective inversion pulse (phase‐modulated hyperbolic secant) is applied in the first heartbeat (HB). Two ECG‐triggered single‐shot images of the same slice are then acquired over the first and second HBs at TIs of TI_1_ and TI_2_, respectively. The delay time between the slice‐selective inversion pulse and the first image (TI_1_) is minimized to reduce the impact of motion. This imaging block is then repeated five times for different slices within the same breathhold.

To reduce the slice mismatch in the presence of residual motion occurring between the inversion and imaging, the ratio of inversion to imaging slice thickness was increased from one to a factor of R_THK_. Furthermore, the five imaging blocks were acquired in a slice‐interleaved fashion using the following slice order (#1, #3, #5, #2, #4) and a slice gap (twice the imaging slice thickness in this work) to minimize slice crosstalk.

To allow for large R_THK_ values and thus improved robustness against motion while minimizing slice crosstalk, recovery HBs were inserted between the third and fourth imaging blocks (between slices #5 and #2, respectively). The number of recovery HBs (N_R_) is adjusted based on each subject's heart rate (HR) to ensure quasi full recovery of the longitudinal magnetization of the last two slices (slices #2, #4) before application of their associated slice‐selective inversion pulses. This adjustment is achieved based on a worst‐case scenario defined as one slice‐selective inversion pulse in the first three imaging blocks (for slices #1, #3, or #5) fully inverted one of its adjacent slices (slices #2 or #4). Considering a normal native myocardial T_1_ at 1.5T (~1200 msec), recovery HBs were added to ensure a minimum delay of five times the native myocardial T_1_ time between the inversion pulses for two adjacent slices (ie, T_RD_ ≥6 sec). As an example, with an HR of 60 bpm, two recovery HBs were used (ie, N_R_ = 2). In the physiological HR range (50–110 bpm), the corresponding nominal breathhold duration is 9–13 sec.

### *T_1_ Map Reconstruction*

T_1_ map reconstruction was performed using an exhaustive search over a signal dictionary. Two models were developed and evaluated for the creation of the signal dictionary using Bloch equations simulation (BS) of the pulse sequence and an IR‐based model. The corresponding reconstructions are thereafter referred to as FAST1‐BS and FAST1‐IR, respectively. Each model was created using a T_1_ range of 1–4000 msec in steps of 1 msec.

#### *BS Model*

The signal dictionary was generated using Bloch equations simulation of FAST1. The signal of each T_1_‐weighted image was simulated as the transversal magnetization at the *k*‐space center (ie, TI_1_ and TI_2_). An initial longitudinal magnetization of 1 was used. T_1_‐dependent slice profiles of the inversion pulse (phase‐modulated hyperbolic secant) and the excitation pulse (nonmodulated Hann‐filtered sinc) were integrated. T_1_‐dependent slice profiles were estimated using Bloch equations simulation of each pulse with a myocardial T_2_ = 45 msec and B0/B1 inhomogeneities [80%,100%]/[–150,150] Hz in steps of 1%/10 Hz. T_1_‐dependent effective flip angles were approximated based on the average longitudinal magnetization over the slice profiles and all simulated T_2_/B0/B1 regimes.

#### *IR Model*

The dictionary was created using a previously proposed normalized one‐parameter model^21^ defined as:

(1) $$S\left(t\right)=1-\left(1+\delta \right)e^{-t/T1},$$

where δ is a constant term representing the inversion factor of the inversion pulse and was determined as ~0.93 using Bloch equations simulation of the inversion pulse in predefined T_1_/T_2_/B0/B1 regimes.^21^

#### *Fitting Process*

The same fitting process was used for both models. Prior to dictionary matching, the signal polarity of the measured signal was restored using a phase sensitive inversion recovery (PSIR) reconstruction approach.^22^ The first image with the shortest TI (TI_1_ = 100 msec) was selected as the reference phase image and was assumed to have "negative" polarity. Based on Bloch equations simulation, this assumption has been shown to be valid for any T_1_ time >172 msec (in the presence of any T_2_ time ≥30 msec and imaging flip angle ≤85°).^21^ Since both signal dictionaries are normalized, the polarity‐restored measured signal S_meas_ was individually scaled to each dictionary entry S_dict_ as:

(2) $$S_{\text{meas}}^{\left(\text{scaled}\right)}=S_{\text{meas}}\cdot \frac{\bar{\left|S_{\text{dict}}\right|}}{\bar{\left|S_{\text{meas}}\right|}},$$

where $\bar{\left|S_{\text{dict}}\right|}$ is the signal amplitude average of a dictionary entry over all TIs (ie, TI_1_ and TI_2_) and $\bar{\left|S_{\text{meas}}\right|}$ is the signal amplitude average of the polarity‐restored measured signal over all TIs (ie, TI_1_ and TI_2_). Dictionary matching was finally performed by minimizing the L2‐norm between $S_{\text{meas}}^{\left(\text{scaled}\right)}$ and each dictionary entry. Graphic processing unit (GPU) implementation of both the dictionary creation and fitting process was developed using the compute unified device architecture (CUDA) (NVIDIA, Quadro K620 2GB) to enable fast T_1_ map reconstruction. For comparison, a standard central processing unit (CPU)‐based implementation was also developed.

#### *HR Correction*

The IR model led to substantial T_1_‐dependence on HR.^21^ Therefore, T_1_ estimates obtained from this model were HR‐corrected as in previous work^21^ and summarized in the Supplementary Materials. No HR correction was performed for the BS model.

### *Monte Carlo Simulation*

Monte Carlo simulation (*N* = 50,000 repetitions) was performed to investigate T_1_/T_2_‐dependent T_1_ accuracy and precision of FAST1‐BS, FAST1‐IR, and standard 5‐(3)‐3 MOLLI. The signal of FAST1 and MOLLI was generated using Bloch equations simulation with the following parameter ranges: T_1_ ([200,2000] msec in steps of 25 msec), and T_2_ ([30,70] msec in steps of 5 msec). Random Gaussian noise was introduced to simulate a typical signal‐to‐noise ratio (SNR) of 50 in the longest TI image of the MOLLI sequence. T_1_ accuracy was calculated as the average over all repetitions of the difference between estimated and actual T_1_ times. T_1_ precision was measured as the standard deviation (SD) over all repetitions of the estimated T_1_ times.

### *Experimental Evaluation*

All imaging experiments were performed using a 1.5T MRI scanner (Magnetom Aera, Siemens Healthcare, Erlangen, Germany). FAST1‐BS and FAST1‐IR were compared with the standard MOLLI sequence (5‐(3)‐3 scheme) in phantom, in healthy volunteers as well as in patients. The in vivo studies were approved by a local Research Ethics Committee (approval number 01/11/12 for the healthy volunteer study and approval number 15/NS/0030 for the patient study), with written informed consent obtained from all participants.

#### *Phantom Experiments*

FAST1‐BS, FAST1‐IR, and MOLLI were initially compared in a phantom (T1MES, Resonance Health, Burswood, WA, Australia) with six vials of different T_1_/T_2_ times representing typical ranges of native and postcontrast myocardial T_1_ times.^23^ Both FAST1 and MOLLI sequences were acquired using the same single‐shot 2D balanced steady‐state free‐precession (bSSFP) readout: repetition time (TR)/ echo time (TE)/ flip angle 2.70 msec/1.12 msec/35°, field of view (FOV) 360 × 306 mm^2^, acquisition matrix 256 × 144, acquired pixel size 1.4 × 2.1 mm^2^, reconstructed pixel size 1.4 × 1.4 mm^2^, slice thickness/gap 8/16mm, GRAPPA acceleration factor 2, partial Fourier factor 7/8, bandwidth 1085 Hz/px, TI_1_ 100 msec. Five slices were acquired using FAST1, while a single slice (the central slice in FAST1) was acquired using MOLLI. Additionally, an IR spin echo (SE) experiment was performed on another day to obtain reference T_1_ times using the following parameters: TE/TR = 15/15000 msec, 15 TIs = [50 msec, 100–900 msec in steps of 100 msec, 1000–5000 msec in steps of 1000 msec], pixel size 1.4 × 1.4 mm^2^, slice thickness 5 mm, and bandwidth 130 Hz/px. Data analysis was performed based on vial‐wise region of interest (ROI) in the common slice unless stated otherwise.

##### *Experiment #1: Influences of T_RD_ and R_THK_*

FAST1 was acquired multiple times using different values of T_RD_ ([4,10] sec in steps of 1 sec) and R_THK_ ([2,8] in steps of 1). A simulated HR of 60 bpm was used for this experiment. The maximum interslice T_1_ variation (max(|Δ_SLICE_T_1_|)) was measured for each set of parameters to identify potential slice crosstalk effects. An empirically optimized pair of T_RD_ (6 sec) and R_THK_ (4) was used for FAST1 in all the following experiments in phantom and in vivo.

##### *Experiment #2: HR sensitivity*

FAST1 and MOLLI were repeated for different simulated HRs ([40,120] bpm in steps of 10 bpm). Mean T_1_ variation across HRs (with respect to T_1_ at 60 bpm) was compared for FAST1‐BS, FAST1‐IR, and MOLLI.

##### *Experiment #3: Characterization of T_1_ accuracy, spatial variability, and repeatability*

FAST1 and MOLLI were each acquired five times using a simulated HR of 60 bpm. T_1_ accuracy, spatial variability, and repeatability were evaluated for FAST1‐BS, FAST1‐IR, and MOLLI. T_1_ accuracy was computed for each vial as the interrepetition average of difference between T_1_ mean in ROI and reference T_1_ obtained in IR SE experiments. T_1_ spatial variability was measured for each vial as the interrepetition average of T_1_ SD in ROI. T_1_ repeatability was evaluated for each vial as the interrepetition SD of T_1_ mean in ROI.

#### *Healthy Volunteer Experiments*

In vivo characterization of native myocardial T_1_ mapping using FAST1‐BS, FAST1‐IR, and MOLLI was performed in nine healthy volunteers (six males, 29 ± 1 years). Both FAST1 and MOLLI were acquired in the short‐axis orientation using the imaging parameters described in the phantom experiments with T_RD_ of 6 sec and R_THK_ of 4. FAST1 was acquired three times to cover the entire LV. To this end, the second and third FAST1 acquisitions were positively and negatively shifted in the slice direction by the employed slice thickness, respectively. This thus resulted in the acquisition of 15 contiguous slices covering the entire LV in a total of three separated breathholds. For comparison, three slices were acquired using MOLLI in another three separated breathholds, matching the three central slices in the first FAST1 acquisition, mimicking a conventional clinical MOLLI protocol.

This entire protocol was performed twice within the same session without subject repositioning to assess the repeatability of in vivo native myocardial T_1_ mapping. Qualitative and quantitative comparisons between both techniques were undertaken in the three common slices (ie, three central slices in the first slice group using FAST1 and three slices using MOLLI).

##### *Qualitative assessment*

No data were discarded for the qualitative assessment. Subjective assessment of map quality for FAST1‐BS, FAST1‐IR, and MOLLI was performed independently by three experienced cardiac MRI readers (M.S.N./J.W./P.D.: >3/4/3 years of cardiac MRI experience) blinded to the techniques. Since blood T_1_ times measured with FAST1 and MOLLI are very different (as FAST1 cannot estimate blood T_1_ due to the in‐flow effect caused by the slice‐selective inversion pulses), the assessment was restrained to the evaluation of map quality only within the myocardium for all techniques to ensure that the readers remain blinded to the acquisition techniques. To this end, endocardial and epicardial contours were manually delineated in the first T_1_‐weighted image of each slice and used to generate a binary mask of the myocardium. The myocardium from each T_1_ map was overlaid to the first T_1_‐weighted image to prevent any bias in the subjective analysis. Each resulting image was then rated using a 4‐point‐scale scoring system defined as: 1, nondiagnostic: artifacts in >50% of AHA (American Heart Association) myocardial segments^24^; 2, fair: artifacts in >1 segments and ≤50% of segments; 3, good: artifacts in 1 segment; 4, excellent: no artifacts. Artifacts were defined as regions of inhomogeneous myocardial T_1_ occurring in areas assessed to be normal tissue.

##### *Quantitative assessment*

All data were visually inspected to detect the presence of severe artifacts or motion among the T_1_‐weighted images. Slices with apparent severe artifacts in any of FAST1‐BS, FAST1‐IR, and MOLLI were discarded from the quantitative analysis of all techniques in that specific subject. Native T_1_ measures, spatial variability, and repeatability of the three techniques were calculated for each AHA myocardial segment.^24^ Native T_1_ measures were calculated as the interrepetition average of the T_1_ mean in a given myocardial segment. Spatial variability was measured as the interrepetition average of the T_1_ SD in a given myocardial segment. Repeatability was evaluated as the interrepetition absolute difference of the T_1_ mean in a given myocardial segment. Subject‐wise T_1_ measures, spatial variability, and repeatability were then computed by averaging the segmental values over all nondiscarded segments for each subject. Segment‐wise T_1_ measures, spatial variability, and repeatability were also computed by averaging the nondiscarded segmental values over all subjects for each myocardial segment. Finally, intersegment variations of native T_1_ measures, spatial variability, and repeatability were calculated as the average over all subjects of the intersegment SD of native T_1_ measures, spatial variability, and repeatability, respectively.

#### *Patient Experiments*

Seventeen consecutive patients (eleven males, 51 ± 17 years) referred for cardiac MRI examination in our center were recruited. The clinical indication for the study included cardiomyopathy (twelve patients), assessment of volumes and function (two patients), assessment for aortopathy (two patients), and investigation of myocarditis (one patient). Native and postcontrast myocardial T_1_ mapping were performed in the short‐axis orientation using FAST1 (15 contiguous slices covering the entire LV in three separated breathholds) and MOLLI (three slices in three separated breathholds, the same as the three central slices in the first slice group of FAST1). Imaging parameters were as described as in the healthy volunteer experiments. Thirteen of these patients (eight males, 51 ± 17 years) received an injection of 0.1 mmol/kg of gadobutrol (Gadovist, Bayer Vital, Leverkusen, Germany) in which postcontrast T_1_ mapping was also performed using FAST1 and MOLLI with the protocol described above.

##### *Qualitative assessment*

Subjective assessment of map quality was performed for native T_1_ maps as described above for the healthy volunteer study.

##### *Quantitative assessment*

Subject‐wise native and postcontrast T_1_ measures were assessed using FAST1‐BS, FAST1‐IR, and MOLLI, as described in the healthy volunteer study.

### *Statistical Analysis*

Data are expressed as mean ± SD. the Kruskal–Wallis test was used to evaluate the null hypothesis that there is no difference in in vivo subjective map quality scores between FAST1‐BS, FAST‐IR, and MOLLI, with statistical significance defined at *P* < 0.05. When the Kruskal–Wallis test found statistical significance, Wilcoxon signed rank tests with Bonferroni correction were performed for each pair of techniques, with statistical significance threshold of *P* < 0.05/3 = 0.0167. Interreader variability was assessed using a two‐way mixed single‐measure intraclass correlation coefficient (ICC).

A one‐way analysis of variance (ANOVA) test was used to evaluate the null hypothesis that there is no difference between the three techniques in terms of myocardial T_1_ times in healthy volunteers, with statistical significance defined at *P* < 0.05. When the ANOVA test found statistical significance, Student's *t*‐tests with Bonferroni correction were performed for each pair of techniques, with statistical significance threshold of *P* < 0.05/3 = 0.0167. The same methodology was used for analysis of myocardial T_1_ spatial variability and T_1_ repeatability in healthy subjects, as well as for analysis of native and postcontrast myocardial T_1_ times in patients.

Pearson correlation and Bland–Altman analyses were also performed between each of the two FAST1 techniques and MOLLI in terms of subject‐wise native/postcontrast myocardial T_1_ times. Bland–Altman 95% limits of agreement were calculated as the mean difference between methods ±1.96 × (SD of differences).

## Results

### *Reconstruction Time*

For a single T_1_ map with a matrix size of 256 × 256, T_1_ map reconstruction of FAST1‐BS/FAST1‐IR took 6.5 sec using CPU implementation and 0.2 sec using GPU implementation. The reconstruction time of an entire FAST1‐BS/FAST1‐IR dataset (ie, five slices) was reduced from 31 sec using CPU implementation to 0.6 sec using GPU implementation. The reconstruction time of three FAST1‐BS/FAST1‐IR datasets for full LV coverage (ie, 15 slices in three slices groups) was reduced from 94 sec using CPU implementation to 1.4 sec using GPU implementation.

### *Monte Carlo Simulation*

**Figure 2:**
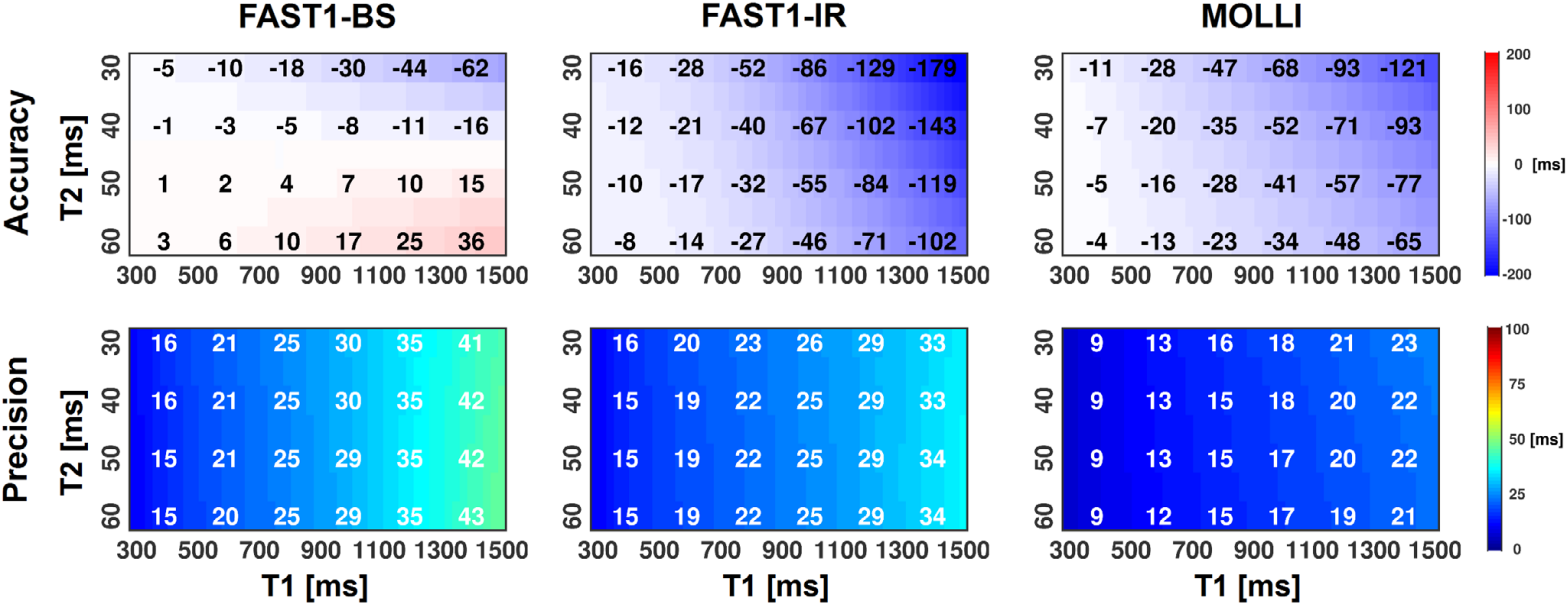
Accuracy and precision of FAST1‐BS, FAST1‐IR, and MOLLI in numerical simulation. FAST1‐BS provided higher accuracy and reduced precision than FAST1‐IR and MOLLI. FAST1‐IR led to reduced accuracy and precision when compared with MOLLI. All techniques were T_2_‐dependent.

Fig. 2 shows the impact of T_2_ on the T_1_ accuracy and precision of FAST1‐BS, FAST1‐IR, and MOLLI. FAST1‐BS led to higher accuracy (mean error: 0.2%) than FAST1‐IR (mean error: 6%) and MOLLI (mean error: 4%). All techniques were T_2_‐dependent. Over the entire studied range of T_1_ and T_2_ times, FAST1‐BS and FAST‐IR led to reduced precision with respect to MOLLI by factors of 1.7 and 1.5, respectively.

### *Phantom Experiments*

##### *Experiment #1: Influences of T_RD_ and R_THK_*

**Figure 3:**
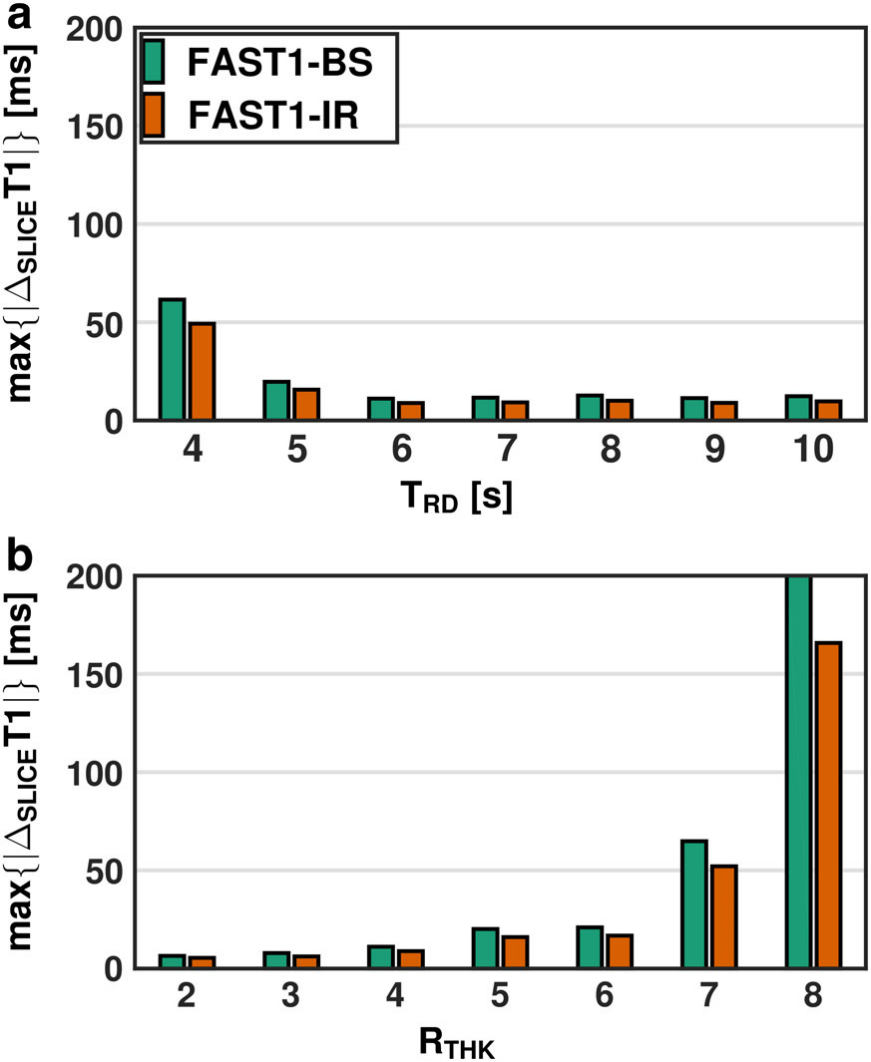
Slice crosstalk using different T_RD_ and R_THK_ using FAST1‐BS and FAST1‐IR in phantom. **(a)** Maximum interslice T_1_ variations among all vials using different T_RD_ from 4 sec to 10 sec and a fixed R_THK_ of 4. With T_RD_ exceeding 6 sec, maximum interslice T_1_ variations were restricted to <13 msec. **(b)** Maximum interslice T_1_ variations among all vials using different R_THK_ from 2 to 8 and a fixed T_RD_ of 6 sec. With R_THK_ not exceeding 4, maximum interslice T_1_ variations were restricted to <11 msec.

Maximum interslice T_1_ variation (max(|Δ_SLICE_T_1_|)) as a function of T_RD_ and R_THK_ is shown in Fig. 3. For both FAST1‐BS and FAST1‐IR, large interslice T_1_ variations of up to 62 msec were observed using a short T_RD_ of 4 sec, while maximum interslice T_1_ variations were substantially reduced to less than 11 msec for T_RD_ exceeding 6 sec. For both techniques, large maximum interslice variations of up to 204 msec were observed using a large R_THK_ of at least 7, while maximum interslice T_1_ variations were substantially reduced to less than 11 msec for R_THK_ not exceeding 4.

##### *Experiment #2: HR sensitivity*

Mean T_1_ variation across HRs obtained using FAST1‐BS, FAST1‐IR, and MOLLI are shown in the Supplementary Materials. All techniques demonstrated minimal HR dependence with mean T_1_ variations across all HRs <13 msec for all vials and all techniques.

##### *Experiment #3: Characterization of T_1_ accuracy, spatial variability, and repeatability*

**Figure 4:**
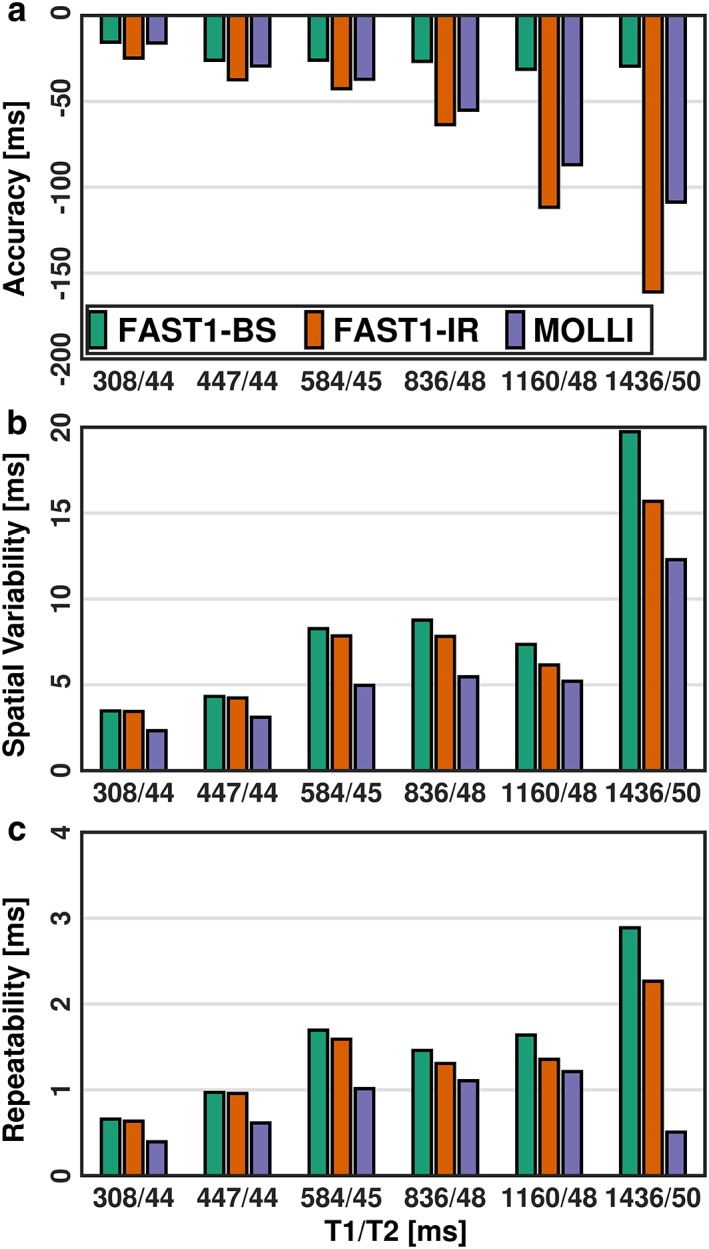
T_1_ accuracy **(a)**, spatial variability **(b)**, and repeatability **(c)** of FAST1‐BS, FAST1‐IR, and MOLLI in phantom. T_1_ error was –26 ± 5 msec vs. –73 ± 53 msec vs. –56 ± 36 msec, T_1_ spatial variability was 9 ± 6 msec vs. 8 ± 4 msec vs. 6 ± 4 msec and T_1_ repeatability was 2 ± 1 msec vs. 1 ± 1 msec vs. 1 ± 0 msec, respectively.

T_1_ accuracy, spatial variability, and repeatability of FAST1‐BS, FAST1‐IR, and MOLLI are shown in Fig. 4. FAST1‐BS, FAST1‐IR, and MOLLI led to T_1_ error of –26 ± 5 msec vs. –73 ± 53 msec vs. –56 ± 36 msec (mean error: 4% vs. 9% vs. 7%), T_1_ spatial variability of 9 ± 6 msec vs. 8 ± 4 msec vs. 6 ± 4 msec (mean penalty factors of FAST1‐BS/IR with respect to MOLLI: 1.5/1.4) and T_1_ repeatability of 1.6 ± 0.8 msec vs. 1.4 ± 0.6 msec vs. 0.8 ± 0.3 msec, respectively.

### *Healthy Volunteer Experiments*

**Figure 5:**
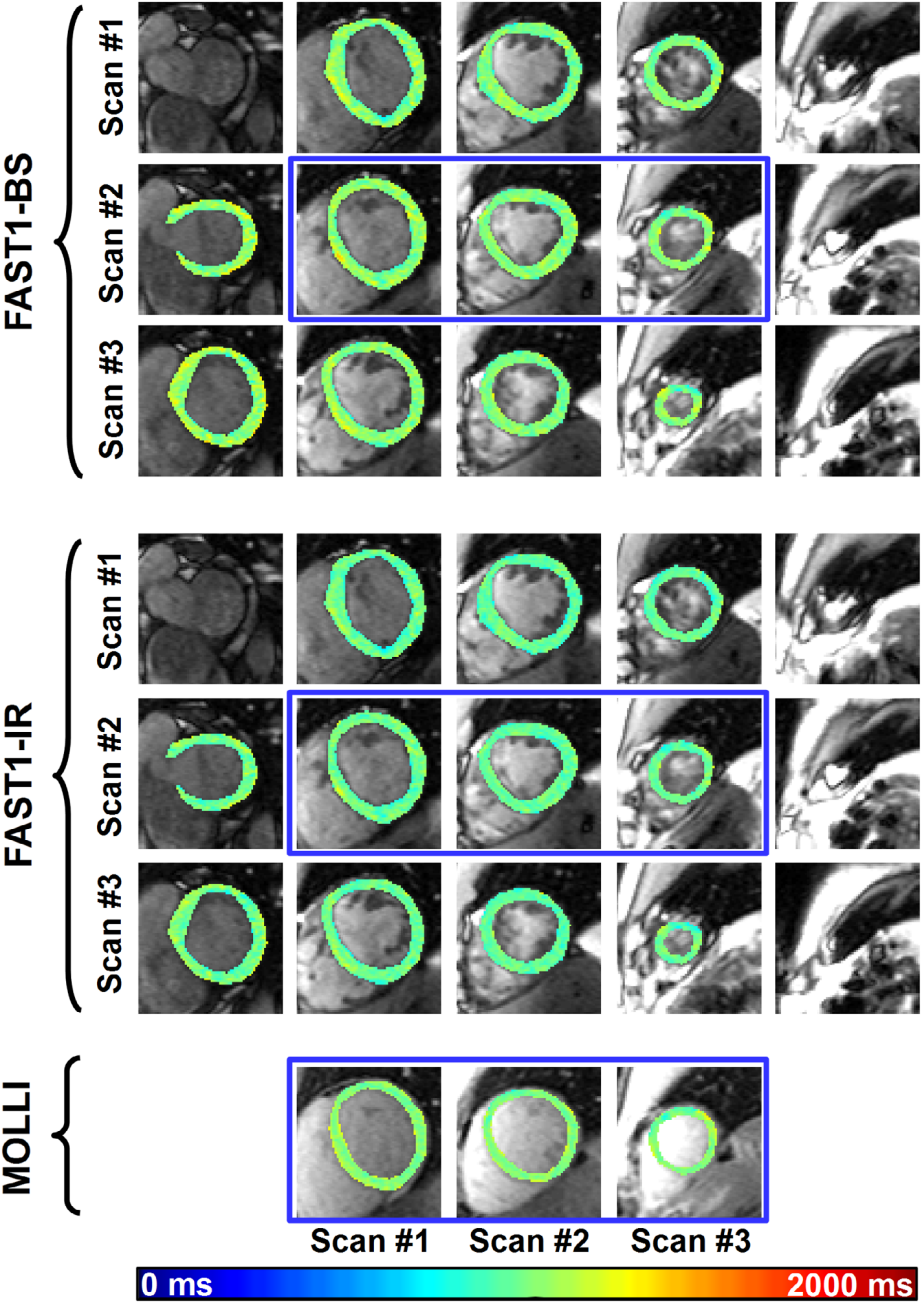
Example native myocardial T_1_ maps measured in one healthy volunteer using FAST1‐BS, FAST1‐IR, and MOLLI. Each row for FAST1‐BS and FAST1‐IR represents one FAST1 acquisition in a separated breathhold. Both FAST1 techniques enabled the acquisition of 15 contiguous slices covering the entire left ventricle in the same time as the acquisition of three slices using MOLLI (ie, 3 breathholds). Note the blue rectangles indicate the three common slice locations in FAST1 and MOLLI.

HR among all healthy volunteers was 66 ± 9 bpm ([51,78] bpm). Breathhold length using FAST1 among all healthy volunteers was 12 ± 1 sec. Example T_1_ maps obtained using FAST1‐BS, FAST1‐IR, and MOLLI in one healthy volunteer are shown in Fig. 5. The three techniques provided similar visual map quality across all slices and myocardial segments. Over all subjects, no statistically significant differences were found between subjective map quality obtained using FAST1‐BS, FAST1‐IR, and MOLLI for each reader (reader #1: 3.3 ± 0.7 vs. 3.6 ± 0.6 vs. 3.4 ± 0.8, respectively, *P* = 0.48; reader #2: 3.4 ± 0.7 vs. 3.6 ± 0.5 vs. 3.6 ± 0.6, respectively, *P* = 0.49; reader #3: 3.6 ± 0.6 vs. 3.9 ± 0.4 vs. 3.7 ± 0.6, respectively, *P* = 0.23; ICC = 0.66).

**Figure 6:**
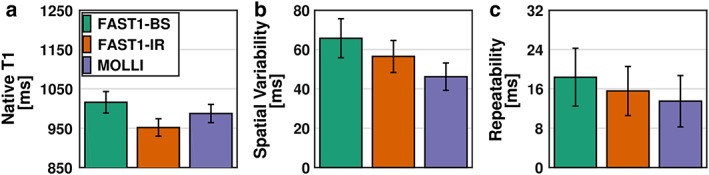
Native myocardial T_1_ times **(a)**, spatial variability **(b)**, and repeatability **(c)** using FAST1‐BS, FAST1‐IR, and MOLLI in healthy volunteers. Average (bar plots) and SD (error bars) over all healthy volunteers are presented. FAST1‐BS, FAST1‐IR, and MOLLI provided different native myocardial T_1_ times (*P* < 0.0001). FAST1‐BS and FAST1‐IR led to higher spatial variability than MOLLI (*P* < 0.001). There were no statistically significant differences between all techniques for T_1_ repeatability (*P* = 0.18).

Among all healthy volunteers, no slices were excluded due to severe artifact level from the data analysis of FAST1‐BS, FAST1‐IR, and MOLLI. Fig. 6 shows the comparison of the three techniques in terms of subject‐wise analysis of native myocardial T_1_ times, spatial variability, and repeatability. Each technique led to different native myocardial T_1_ times (FAST1‐BS: 1016 ± 27 msec, FAST‐IR: 952 ± 22 msec, MOLLI: 987 ± 23 msec, *P* < 0.0001) and spatial variability (FAST1‐BS: 66 ± 10 msec, FAST‐IR: 57 ± 8 msec, MOLLI: 46 ± 7 msec, *P* < 0.001). Spatial variability increases of FAST1‐BS and FAST1‐IR with respect to MOLLI were by factors of 1.4 and 1.2, respectively. There were no statistically significant differences between all techniques in terms of T_1_ repeatability (FAST1‐BS: 18 ± 6 msec, FAST1‐IR: 16 ± 5 msec, MOLLI: 14 ± 5 msec, *P* = 0.18).

**Figure 7:**
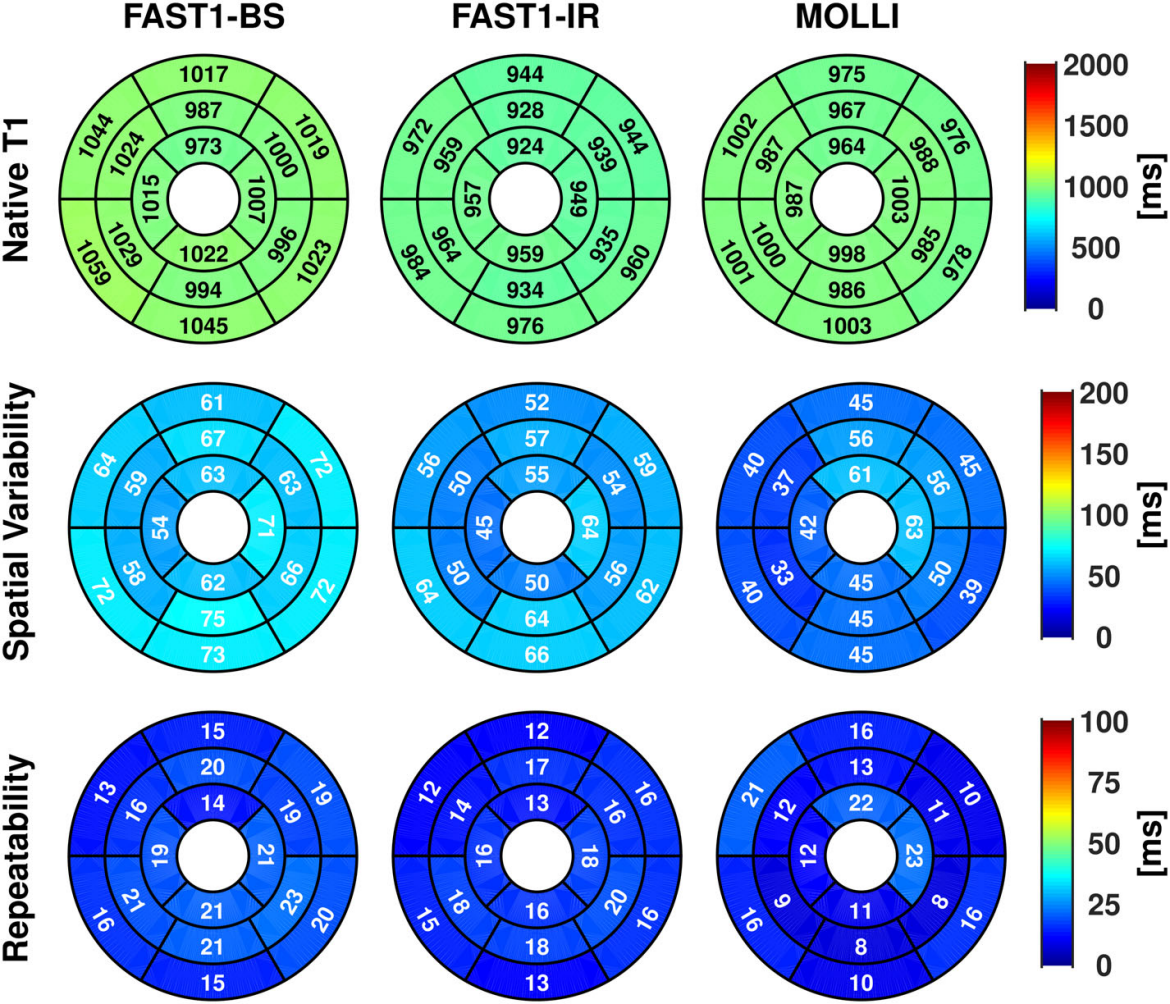
Segment‐wise native myocardial T_1_ measures, spatial variability, and repeatability using FAST1‐BS, FAST1‐IR, and MOLLI in healthy volunteers. There were no statistically significant differences between all techniques in terms of segmental variations of native T_1_ measures (*P* = 0.20), spatial variability (*P* = 0.32), and repeatability (*P* = 0.58).

Myocardial segment‐based analysis is shown in Fig. 7. There were no statistically significant differences between FAST1‐BS, FAST1‐IR, and MOLLI in terms of segmental variations of native T_1_ measures (31 ± 9 msec vs. 25 ± 7 msec vs. 24 ± 8 msec, respectively, *P* = 0.20), segmental variations of T_1_ spatial variability (13 ± 2 msec vs. 11 ± 2 msec vs. 12 ± 4 msec, *P* = 0.32), and segmental variations of T_1_ repeatability (13 ± 6 msec vs. 11 ± 5 msec vs. 11 ± 6 msec, *P* = 0.58).

### *Patient Experiments*

**Figure 8:**
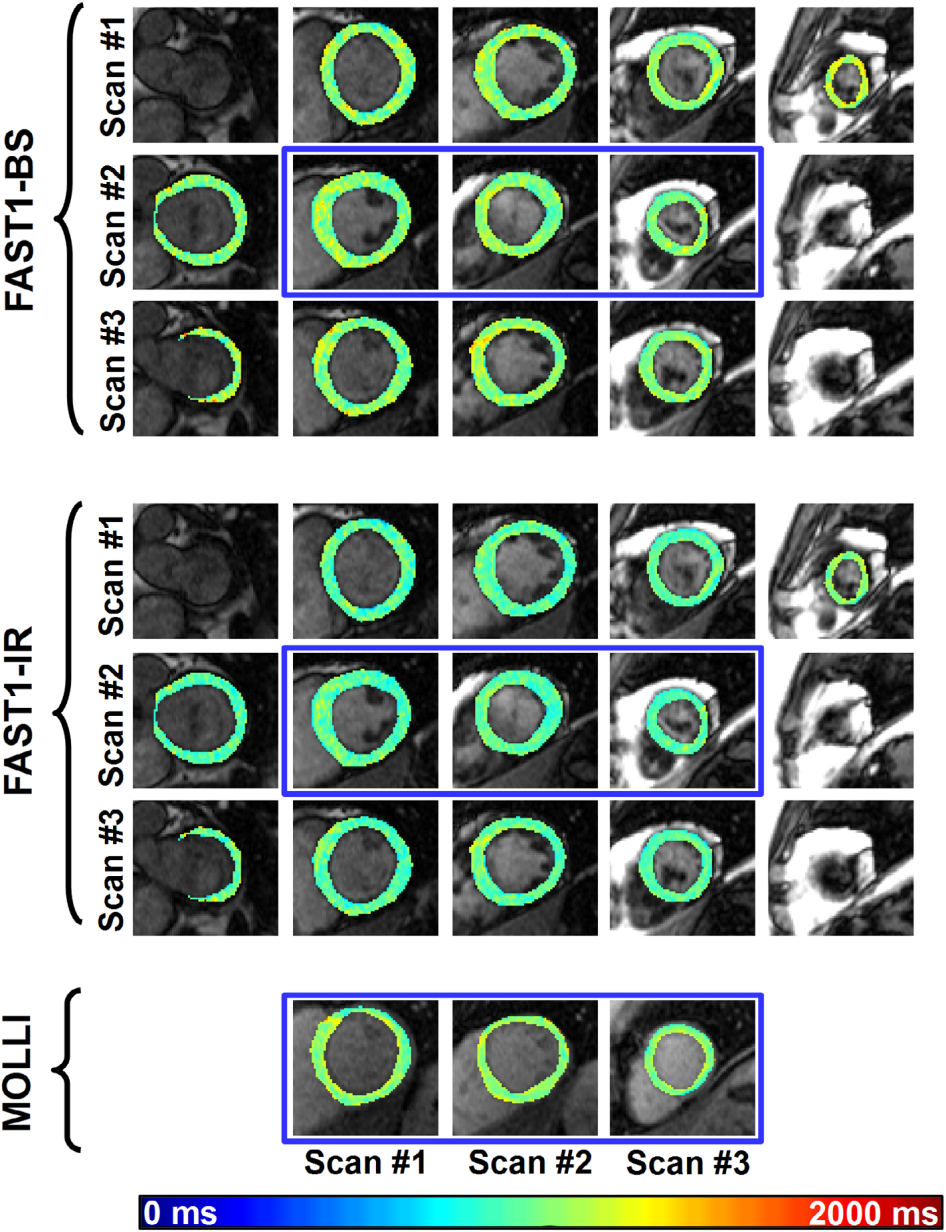
Example native myocardial T_1_ maps obtained using FAST1‐BS, FAST1‐IR, and MOLLI in a 31‐year‐old male patient admitted for suspected myocarditis. Each row for FAST1‐BS and FAST1‐IR represents one FAST1 acquisition in a separated breathhold. Both FAST1 techniques enabled the acquisition of 15 contiguous slices covering the entire left ventricle in the same time as the acquisition of three slices using MOLLI (ie, three breathholds). Note that the blue rectangles indicate the three common slice locations in FAST1 and MOLLI.

**Figure 9:**
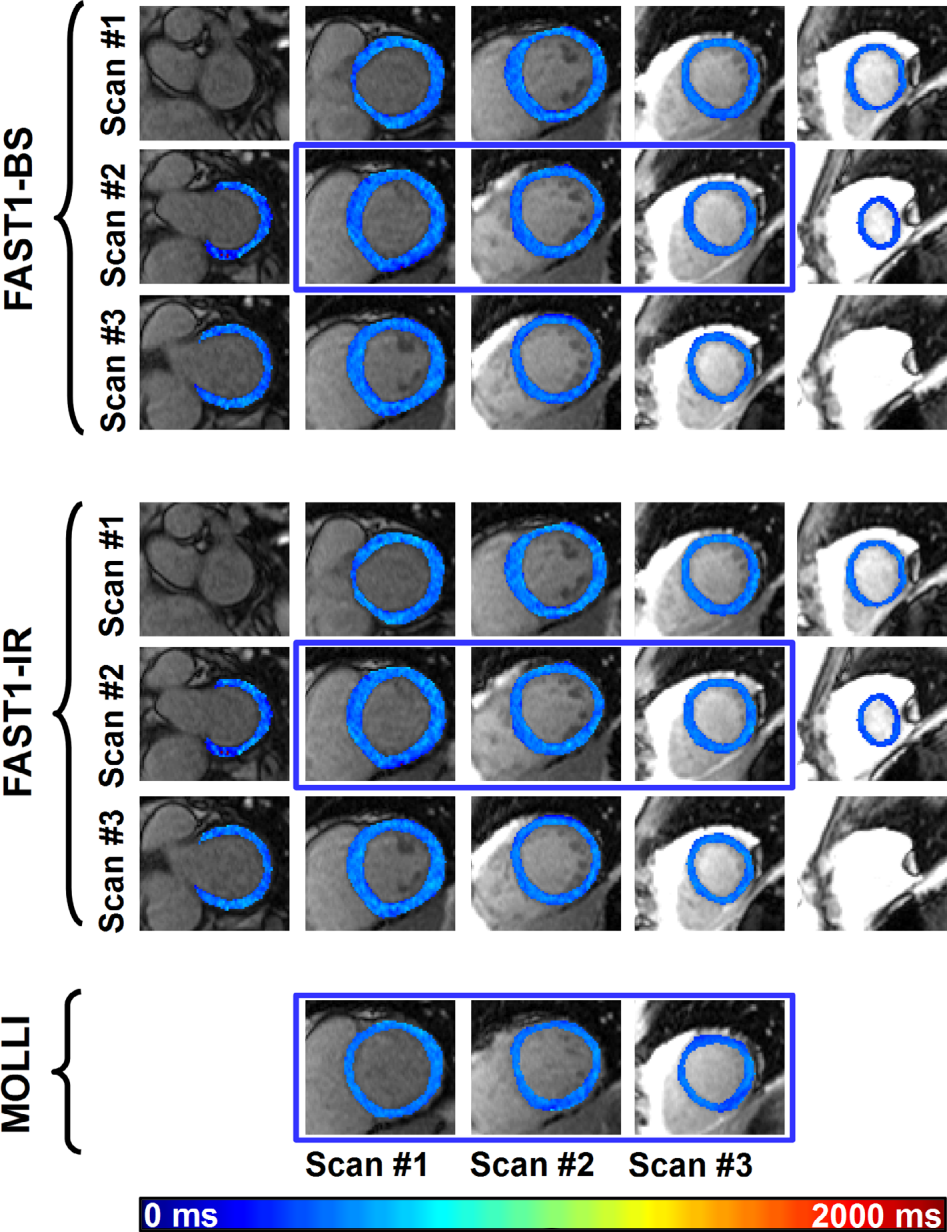
Example postcontrast myocardial T_1_ maps obtained using FAST1‐BS, FAST1‐IR, and MOLLI in a 31‐year‐old male patient admitted for suspected myocarditis. Each row for FAST1‐BS and FAST1‐IR represents one FAST1 acquisition in a separated breathhold. Both FAST1 techniques enabled the acquisition of 15 contiguous slices covering the entire left ventricle in the same time as the acquisition of three slices using MOLLI (ie, three breathholds). Note the blue rectangles indicate the three common slice locations in FAST1 and MOLLI.

HR among all patients was 68 ± 12 bpm ([52,92] bpm). Breathhold length using FAST1 among all patients was 12 ± 2 sec. Figs. 8 and 9 show example native and postcontrast T_1_ maps obtained using FAST1‐BS, FAST1‐IR, and MOLLI in a 31‐year‐old male patient admitted for suspected myocarditis. Over all patients, FAST1‐BS resulted in higher subjective map quality than MOLLI (reader #1: 3.7 ± 0.5 vs. 3.4 ± 0.8, *P* = 0.004; reader #2: 3.8 ± 0.5 vs. 3.5 ± 0.7, *P* = 0.002; reader #3: 3.4 ± 0.8 vs. 3.2 ± 0.8, *P* = 0.20), although these differences only reached statistical significances for readers #1 and #2. FAST1‐IR resulted in higher subjective map quality than MOLLI (reader #1: 3.7 ± 0.5 vs. 3.4 ± 0.8, *P* = 0.003; reader #2: 3.8 ± 0.5 vs. 3.5 ± 0.7, *P* = 0.006; reader #3: 3.6 ± 0.6 vs. 3.2 ± 0.8, *P* = 0.0005). The interreader ICC was 0.58.

No slices in FAST1‐BS and FAST1‐IR were found with a severe artifact level, while a total of eight slices in MOLLI were identified with severe respiratory motion artifacts (8.9% of 90 slices) and subsequently discarded for all techniques for the quantitative analysis. Native myocardial T_1_ times using FAST1‐BS, FAST1‐IR, and MOLLI were 1057 ± 50 msec, 987 ± 42 msec, and 1036 ± 39 msec, respectively (*P* < 0.0001). On the other hand, there were no statistically significant differences between all techniques for postcontrast T_1_ times (469 ± 54 msec, 455 ± 52 msec and 454 ± 49 msec, respectively, *P* = 0.72).

**Figure 10:**
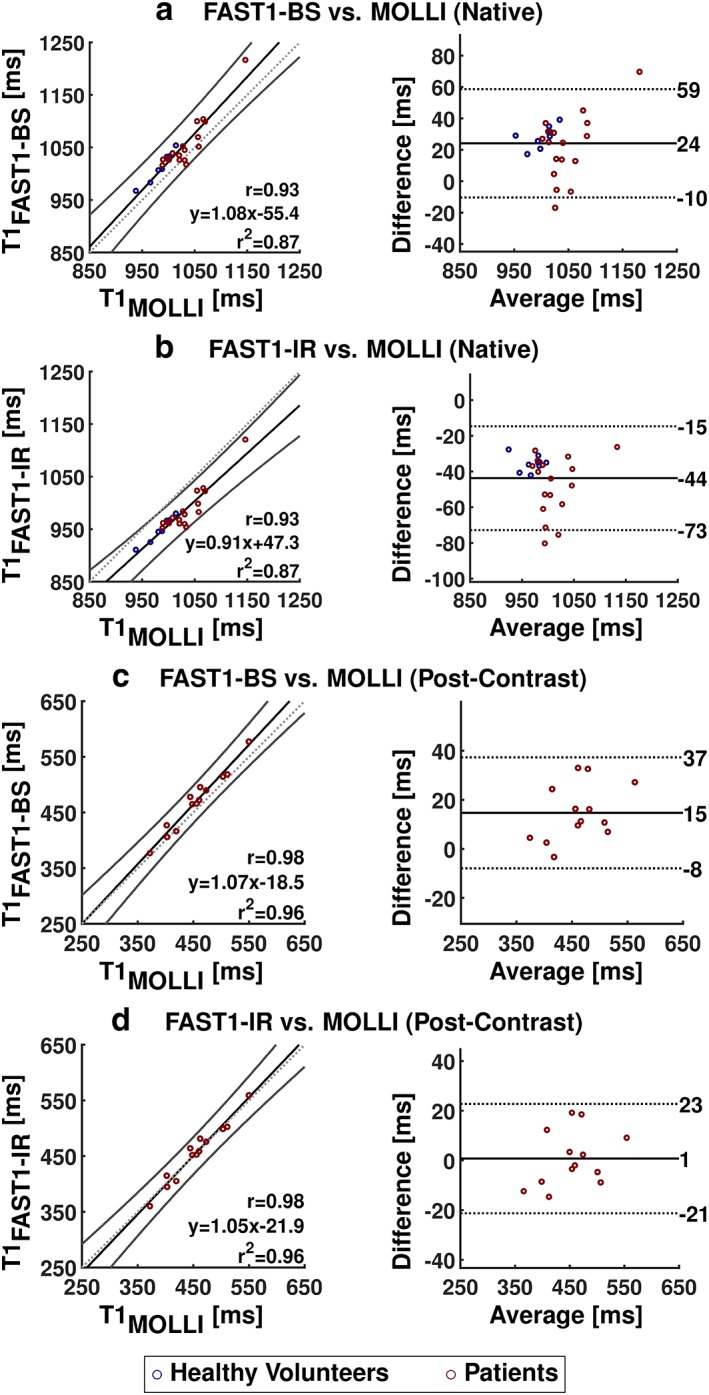
Pearson correlation and Bland–Altman analyses between FAST‐BS and MOLLI **(a,c)** as well as between FAST1‐IR and MOLLI **(b,d)** for both native and postcontrast myocardial T_1_ times measured in both healthy volunteers and patients. In Pearson correlation analysis plots, confidence interval (solid lines) and identity line (y = x, dashed line) are also plotted besides the linear regression line (solid line). FAST1‐BS and FAST1‐IR yielded highly linearly correlated T_1_ times with MOLLI (Pearson correlation coefficient = 0.93 for native and 0.98 for postcontrast myocardial T_1_ times).

Pearson correlation and Bland–Altman analyses of subject‐wise native and postcontrast myocardial T_1_ times (in healthy volunteers and patients) between FAST1‐BS and MOLLI as well as between FAST‐IR and MOLLI are shown in Fig. 10. FAST1‐BS/FAST1‐IR were highly linearly correlated with MOLLI for both native and postcontrast myocardial T_1_ estimates (Pearson correlation coefficient = 0.93/0.93 with *P* < 0.001 for native and 0.98/0.98 with *P* < 0.001 for postcontrast). For native myocardial T_1_ estimates, FAST1‐BS and FAST1‐IR led to a bias of 24 ± 18 msec and –44 ± 15 msec with respect to MOLLI, respectively, with a narrow width of 95% limits of agreement (70 msec and 59 msec, respectively). For postcontrast myocardial T_1_ estimates, FAST1‐BS and FAST1‐IR led to a bias of 15 ± 12 msec and 1 ± 11 msec with respect to MOLLI, respectively, with a narrow width of 95% limits of agreement (46 msec and 45 msec, respectively).

## Discussion

FAST1 enables multislice myocardial T_1_ mapping in one breathhold and full LV coverage in three breathholds. Two FAST1 reconstructions were developed, characterized, and compared with MOLLI in simulation, phantom, healthy volunteers, and patients. The resulting native and postcontrast myocardial T_1_ times obtained using FAST1‐BS/FAST1‐IR and MOLLI showed strong linear correlation. In comparison to MOLLI, FAST1‐BS/FAST1‐IR led to a 5‐fold increase of spatial coverage within the same time frame, limited precision penalty, and no statically significant difference of repeatability.

The sequence parameters T_RD_ and R_THK_ were optimized to ensure the robustness of the sequence in the presence of potential slice crosstalk due to cardiac/respiratory motion and imperfect slice profile with side lobes. T_RD_ was optimized based on normal native myocardial T_1_ times at 1.5T. The application of FAST1 at different field strengths or for different tissues of interest may require adjustment of this parameter. The optimized R_THK_ was directly related to the employed imaging slice thickness and slice gap. In this work, R_THK_ of 4, ie, an inversion slice thickness of 32 mm, was found suitable to account for elevated HR. A slice gap of twice the imaging slice thickness was used in this work to avoid gaps or overlaps between slice groups within different breathholds, as we aimed to achieve full LV coverage in three separated breathholds. This parameter should be carefully selected with respect to R_THK_, the employed imaging slice thickness, and the slice profile of the slice‐selective inversion pulse in order to avoid slice crosstalk. The development of a slice‐selective inversion pulse with improved slice profile could, however, increase the flexibility of the sequence with respect to these parameters.

In this work, 15 contiguous slices were acquired, which resulted in a spatial coverage of 120 mm in the long‐axis dimension. As most hearts are less than 100 mm in the long‐axis dimension, slightly reduced coverage may be sufficient for most patients. Although not directly demonstrated in this work, two different strategies could be envisioned to reduce spatial coverage. First, reduced slice thickness/slice gap of 7/14 mm could be used, leading to a total spatial coverage of 15 × 7 mm = 105 mm. Reducing the slice gap could increase the sensitivity of FAST1 to slice crosstalk. However, a small slice gap reduction of 2 mm (from 16 mm to 14 mm) as proposed in this alternative strategy is expected to have minimal impact on slice crosstalk. Reducing the spatial resolution would reduce the SNR in the T_1_‐weighted images, and thus the precision of T_1_ estimates. However, the relative precision penalty of FAST1 with respect to MOLLI is expected to be SNR‐independent based on our previous work using a two‐heartbeat T_1_ mapping scheme (see Ref. 21, Supplementary Material 6). Alternatively, reduced spatial coverage could be achieved by acquiring only four slices per FAST1 scan (instead of five), which would result in a total spatial coverage of 12 × 8 mm = 96 mm. This could be achieved by discarding the first two heartbeats (ie, slice #1), which would also shorten the required breathholds.

FAST1‐BS was more accurate than FAST‐IR and MOLLI, which is likely due to its more accurate modeling of the imaging pulses. FAST1‐BS was found to be HR‐independent. FAST1‐IR required the use of a novel HR correction approach to reduce its original HR‐dependence.^21^ The HR correction designed for FAST1‐IR was calibrated using phantom data to provide a method easily translatable to a different scanner. Therefore, it is possible that this model may be suboptimal when applied in vivo. However, the high correlation between FAST1‐IR and MOLLI suggests that this correction performed relatively well. Furthermore, FAST1‐BS and FAST1‐IR may be sensitive to myocardial blood flow due to the use of slice‐selective inversion pulses.^25^ As FAST1 and MOLLI use analogous acquisition schemes, FAST1‐BS and FAST1‐IR may also be sensitive to magnetization transfer, which was shown to be the main contributor for the underestimation of in vivo native myocardial T_1_ time using MOLLI.^26^ FAST1‐BS may thus have an advantage over FAST1‐IR, as it could enable the integration of the magnetization transfer effect in the creation of the signal dictionary.^27^

T_1_ spatial variability is an important criterion for clinical applicability of any T_1_ mapping technique. FAST‐IR led to an increase of T_1_ spatial variability by a factor of 1.2 for in vivo native myocardial T_1_ times when compared with MOLLI. This result is in the same order as those reported for the widely used ShMOLLI technique compared with MOLLI for native myocardial T_1_ mapping at 1.5T.^6^ Since ShMOLLI usually only considers the first five T_1_‐weighted images only for native myocardial T_1_ setting, this suggests that long TI T_1_‐weighted images have reduced contributions to the precision of T_1_ estimates due to their reduced T_1_‐weighted contrast. FAST1‐BS leads to slightly higher increase of T_1_ spatial variability (by a factor of 1.4 when compared with MOLLI), but has higher accuracy, as discussed above.

Although FAST1 is based on inversion pulses, this sequence could be modified to use saturation pulses instead. Myocardial T_1_ mapping using two images only and a saturation recovery approach has been previously proposed using the arrhythmia insensitive rapid (AIR) T_1_ mapping technique, although this technique only enabled the acquisition of one T_1_ map per breathhold.^28^ A saturation recovery‐based FAST1 sequence could be developed using slice‐selective saturation pulses or the acquisition of all nonmagnetization prepared images at the beginning of the scan. Nevertheless, AIR was shown to considerably increase the spatial variability of native myocardial T_1_ mapping by a factor of 2.5 when compared with MOLLI.^29^ The proposed IR‐based FAST1 approach resulted in a limited increase of spatial variability for native myocardial T_1_ mapping by a factor of 1.4 (FAST1‐BS) and 1.2 (FAST1‐IR) when compared with MOLLI. Furthermore, the HR‐independence of FAST1‐BS and FAST1‐IR also suggests their insensitivity to arrhythmia, as only two images are acquired per slice. Although not directly demonstrated in this study, these findings suggest that an IR‐based FAST1 approach may have substantial advantages over a saturation recovery‐based FAST1 approach.

In this work, only the short‐axis orientation was investigated to minimize the sensitivity to the partial volume effect compared with the other orientations. However, the short‐axis orientation is suboptimal for imaging at the apical level, and the use of an additional long‐axis slice may be beneficial if mapping of the apex is intended.

Motion correction was not performed for FAST1 and MOLLI. Since each T_1_ map is reconstructed from only two images in FAST1 and eight images in MOLLI, it is possible than FAST1 provide better native image registration, which could have potentially explained the slightly reduced T_1_ map quality of MOLLI with respect to FAST1 in patients. Existing image registration algorithms may provide different performance based on the number of T_1_‐weighted images and the presence of an in‐flow effect in the LV blood pool such as in FAST1. Therefore, to prevent any bias between the techniques induced by the choice of the image registration algorithm, this step was not applied in this work. Nevertheless, image registration algorithms were shown to improve myocardial T_1_ map quality.^30^, ^31^ Therefore, the development of an image registration step in the FAST1 reconstruction will be investigated in future work.

FAST1 does not allow for quantification of blood T_1_ times due to the in‐flow effect caused by the slice‐selective inversion pulse. Therefore, FAST1 cannot be directly applied for extracellular volume (ECV) quantification. The combination of FAST1 with an additional mid‐ventricular T_1_ map acquired using nonselective inversion for blood T_1_ quantification could enable multislice ECV mapping with FAST1 and will be investigated in future work.

This work was performed at 1.5T. Future work will investigate the feasibility of FAST1 at 3T with the potential benefit for scar assessment in patients with chronic myocardial infarction.^5^ Due to the longer native myocardial T_1_ times at 3T, a longer T_RD_ may be necessary to achieve nearly full recovery of the longitudinal magnetization. The combination of FAST1 with a gradient recalled echo (GRE) readout could also be beneficial to reduce off‐resonance artifacts at higher fields.^32^, ^33^

This work has several limitations. First, the approximation of the slice profile was approximated by one flip angle only. More advanced modeling could be considered in future work to better represent the nonlinear signal response to the flip angle.^33^ Second, postcontrast myocardial T_1_ mapping using FAST1 was not characterized in healthy volunteers. However, the phantom and patient experiments demonstrated the feasibility of postcontrast myocardial T_1_ mapping using FAST1. Third, no statistical analysis was performed for the phantom study, as only six vials with realistic myocardial T_1_ times were available, thus limiting the available power. However, the trend observed in the phantom experiments was confirmed in both numerical simulations and in vivo studies. Finally, the patient study was used for studying feasibility. Further studies in larger patient cohorts are now warranted.

In conclusion, FAST1 enables myocardial T_1_ mapping with full LV coverage in three separated breathholds. In comparison with MOLLI, FAST1‐BS and FAST1‐IR yield a 5‐fold increase of spatial coverage, limited penalty of T_1_ precision/spatial variability, no significant difference of T_1_ repeatability, and highly correlated T_1_ times. FAST1‐IR provides improved T_1_ precision/spatial variability but reduced accuracy when compared with FAST1‐BS.

## Supporting information


**Appendix S1**: Supplementary Materials
