# Supplementary figures and images for: FASt single‐breathhold 2D multislice myocardial T1 mapping (FAST1) at 1.5T for full left ventricular coverage in three breathholds
Source: J Magn Reson Imaging. 2019 Jul 24;51(2):492–504. doi: 10.1002/jmri.26869 (PMC6954880; doi:10.1002/jmri.26869)

*
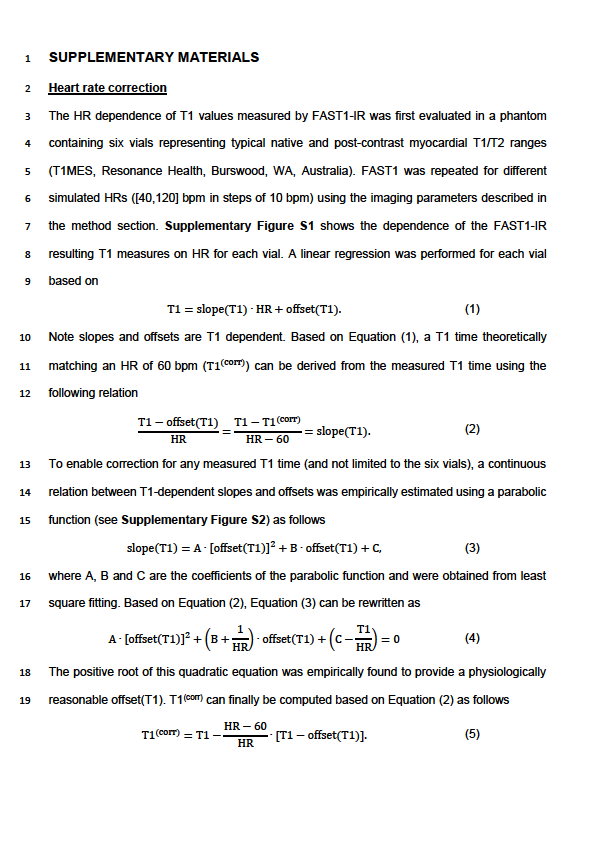
*

Supplement: Supplementary file 1 — Appendix S1: Supplementary Materials [file JMRI-51-492-s001.doc]
